# Biological insertion of computationally designed short transmembrane segments

**DOI:** 10.1038/srep23397

**Published:** 2016-03-18

**Authors:** Carlos Baeza-Delgado, Gunnar von Heijne, Marc A. Marti-Renom, Ismael Mingarro

**Affiliations:** 1 Departament de Bioquímica i Biologia Molecular, ERI BioTecMed, Universitat de València. E-46100 Burjassot, Spain; 2 Dept. of Biochemistry and Biophysics and Science for Life Laboratory, Stockholm University, 10691 Stockholm, Sweden; 3 CNAG-CRG, Centre for Genomic Regulation (CRG), Barcelona Institute of Science and Technology (BIST), 08028 Barcelona, Spain; 4 Universitat Pompeu Fabra (UPF), 08002 Barcelona, Spain; 5 Institució Catalana de Recerca i Estudis Avançats (ICREA), 08010 Barcelona, Spain

## Abstract

The great majority of helical membrane proteins are inserted co-translationally into the ER membrane through a continuous ribosome-translocon channel. The efficiency of membrane insertion depends on transmembrane (TM) helix amino acid composition, the helix length and the position of the amino acids within the helix. In this work, we conducted a computational analysis of the composition and location of amino acids in transmembrane helices found in membrane proteins of known structure to obtain an extensive set of designed polypeptide segments with naturally occurring amino acid distributions. Then, using an *in vitro* translation system in the presence of biological membranes, we experimentally validated our predictions by analyzing its membrane integration capacity. Coupled with known strategies to control membrane protein topology, these findings may pave the way to *de novo* membrane protein design.

Transmembrane (TM) helices are the building blocks of the vast majority of integral membrane proteins. To perform their biological functions, integral membrane proteins must first insert their TM segments into the membrane in a helical conformation, and then acquire a defined three-dimensional structure by assembling their TM helices. In eukaryotic cells, the insertion and assembly of membrane proteins is mediated by the concerted action of a translating ribosome and the endoplasmic reticulum (ER) translocon, which allows lateral integration of TM helices into the ER membrane^1^. Although it is well accepted that thermodynamically favorable partitioning of TM helices from the translocon into the more hydrophobic (lipidic) environment is important at the insertion stage^2^,^3^, the limits for the insertion of TM helices with naturally occurring amino acid distributions has not been systematically explored.

The minimum hydrophobic length necessary to form a TM helix has been investigated using model membrane-inserted hydrophobic peptides^4^. These results show that for alternating Leu and Ala peptides (which have a hydrophobicity typical of natural TM helices), 13 consecutive residues is the minimum necessary to adopt a predominantly TM disposition in synthetic bilayers with a biologically relevant thickness. More recently, TM disposition of poly-Leu sequences were analyzed using synthetic peptides and oriented phospholipid bilayers, *in vitro* insertion into microsomal membranes and molecular dynamics simulations^5^. Sequences with either blocks of or dispersed hydrophobic residues have also been analyzed using similar methods^6^. The picture that emerges from these studies is that lipid bilayers adapt to TM helices as short as 10–12 leucines. However, TM helices in native membrane proteins vary significantly in length, being 24.0 (±5.6) amino acids long on average^7^.

How exactly a particularly short TM helix responds to the surrounding lipid bilayer will depend not only on the nature of the lipids with which it is in contact but also on its amino acid composition and the distribution of amino acids along the helix. While the studies cited above are all based on model hydrophobic sequences composed of only a few different kinds of amino acids, TM helices of amino acid composition that more closely matches natural TM helices need to be studied to lay a foundation for more advanced TM helix designs.

Here we used a database including 792 TM helices from membrane proteins with known three-dimensional structure and topology to generate a collection of random sequences with naturally occurring TM compositions of different lengths. The membrane insertion efficiency of these sequences were predicted computationally and systematically examined experimentally using a microsomal *in vitro* expression system. The results reveal a correlation between the predictions and the experimental data, but also highlight the importance of local residue positioning, particularly with respect to the presence of proline residues and putative salt bridges within the TM segment.

## Results

### Predicted insertion capacity for designed sequences

**Figure 1:**
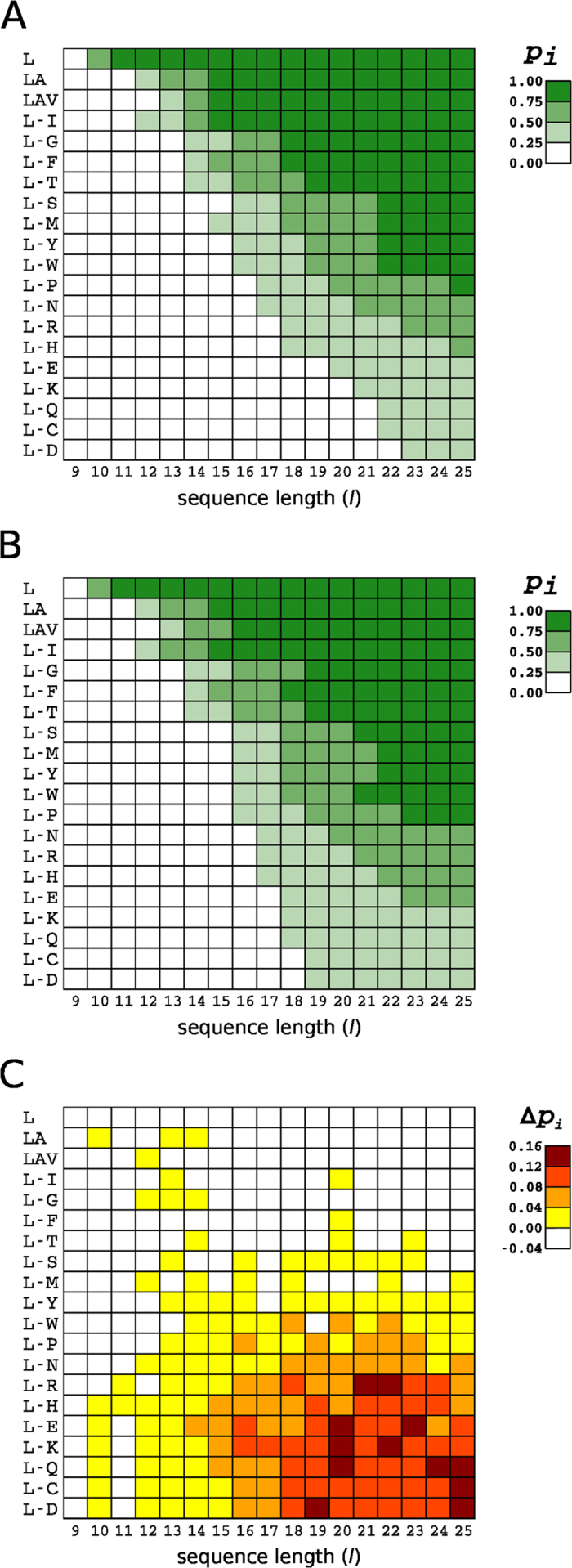
Predicted membrane insertion efficiencies for computationally designed sequences. (**A**) Probability of insertion (*p*_*i*_) for a series of computationally designed TM segments of different lengths. First row corresponds to the predictions of *p*_*i*_ values for polyleucine stretches of different lengths (*l*). Each row in descending order represents the inclusion of a specific amino acid to the TM segment composition used in the previous row. The row order is derived from the prevalence for each amino acid type in TM helix composition in a previous structure-based statistical analysis^7^. (**B**) Similar to (**A**) but including in the computational design information of the position-dependent distribution for each amino acid type in TM helices^7^. (**C**) Differences between the position-defined (**B**) *p*_*i*_ values and those obtained for the computed sequences using only amino acid composition constraints (**A**).

The membrane insertion efficiency of the computationally designed sequences (see Methods and Supplementary Information Fig. S1 for details of the design procedure) was predicted using the experimentally based ΔG Prediction Server (http://dgpred.cbr.su.se). In an initial screen, the insertion efficiency of polyleucine segments of different lengths (*l*, from 9 to 25 residues) was calculated. Stretches of 11 or more leucine residues were predicted to be fully inserted, while 9 consecutive leucines was not enough to be inserted and 10 leucines resulted in a probability of insertion (*p*_*i*_) of 0.59 (Fig. 1A, first row). As expected, these results were in excellent agreement with the previous experimental data^5^ that was used to construct the ΔG Predictor. These extremely short sequences would likely provoke the adaptation of the surrounding bilayer to reduce the putative hydrophobic mismatch by changes in the lipid order parameters in the peptide neighborhood, according to molecular dynamics simulations^5^.

We thus began by testing computationally designed segments with more variable amino acid composition, initially formed by leucine and alanine residues, which are the two most prevalent residues in TM helices. Sets of 1,000 sequences from 9 to 25 residues long were computed with the leucine-alanine relative ratio (58.2%Leu-41.8%Ala) found in TM helices in membrane proteins of known structures^7^. At least 13 residues were needed to obtain a significant level of predicted insertion (Fig. 1A second row), which was in good agreement with what it was found for alternating leucine and alanine peptides in lipid vesicles^4^. Subsequently, we included one by one the rest of the 20 natural amino acids following the order of prevalence found in native membrane proteins^7^. For instance, sets of 1,000 sequences formed by leucines, alanines and valines were generated using 42.6% Leu, 30.6% Ala and 26.8% Val residues, for each sequence length (SI Table 1). As expected, sequence sets including less hydrophobic residues needed longer segments to achieve insertion *p*_*i*_ values greater than 0.5 (Fig. 1A). Interestingly, when the less abundant lysine, glutamine, cysteine and aspartate residues were included at their naturally observed frequencies, the algorithm predicted that these computationally designed sequences were not expected to be efficiently inserted into biological membranes through the ER translocon. However, some natural sequences with these precise compositions are successfully inserted *in vivo*. Therefore, in line with previous findings^3^, we hypothesized that not only the amino acid composition is relevant for TM insertion, but that the actual position of the amino acid residues with respect the center of the TM segment could also have an important influence on the insertion process.

To address this question, we changed our computational design algorithm to reflect the amino acid frequency values stratified at different position within a TM segment, taking as a reference the TM center^7^. Again, sets of 1,000 sequences were designed, using this position-dependent distribution of residues in natural TM helices, and their *p*_*i*_ values were predicted (Fig. 1B). The predicted insertion efficiency for sets with higher proportion of hydrophilic residues increased significantly when residue position constrains were included in the computational design. Hence, series of position-defined sequences including arginine, histidine, glutamate, lysine, glutamine, cysteine and aspartate were predicted to insert more efficiently at any given segment length than the previous sets based only on the global TM helix residue composition. The differences between the two data sets were most evident for TM sequences that include the less prevalent, more hydrophilic residues (Fig. 1C).

### Correlation between predicted and experimentally determined insertion efficiencies

**Figure 2:**
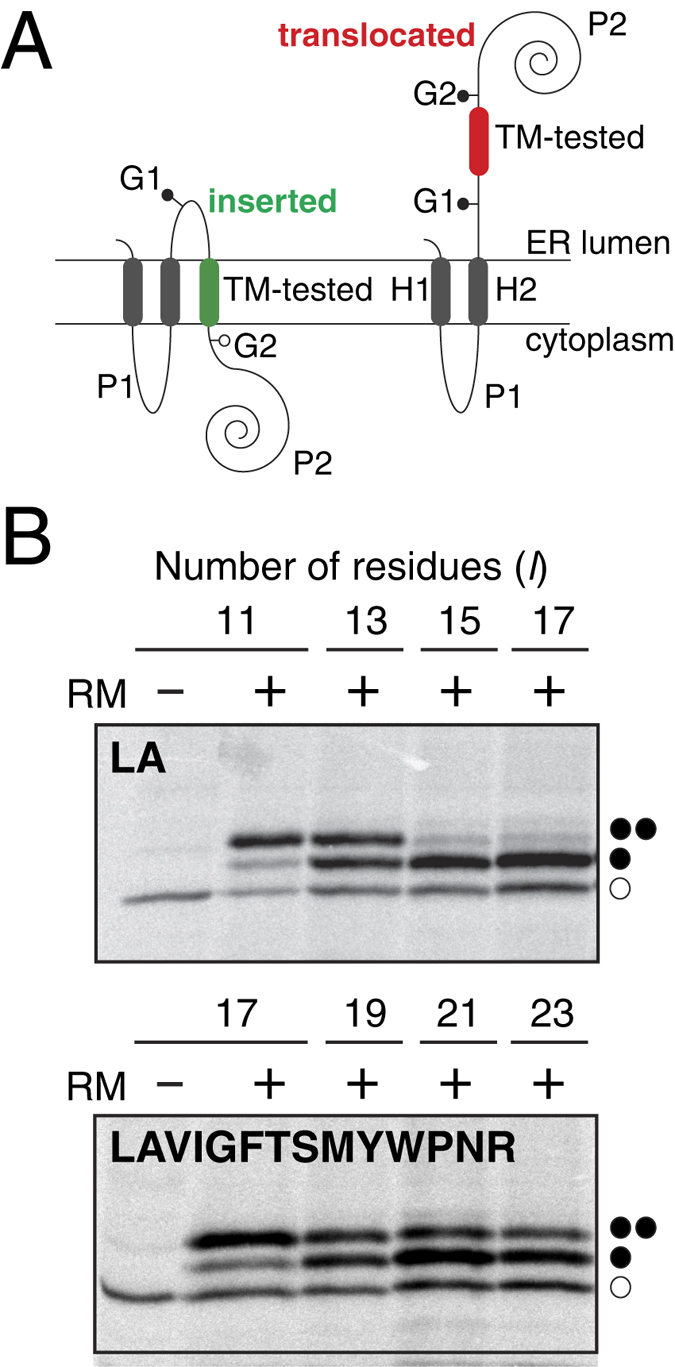
Integration of computationally designed TM segments into microsomal membranes. (**A**) Schematic of the engineered leader peptidase (Lep) model protein. Lep, consisting of 2 TM segments (H1 and H2) and a large luminal domain (P2), inserts into rough microsomes in an N_lum_-C-_lum_ orientation. Computationally designed TM sequences were engineered into the P2 domain with flanking glycosylation sites (G1 and G2). For sequences that integrate into the membrane (green), only the G1 site is glycosylated (left), whereas both G1 and G2 are modified for sequences (red) that do not integrate into the membrane (right). (**B**) *In vitro* translation in the presence (+) or absence (−) of rough microsomes (RM) of computationally designed TM sequences of different length (*l*) composed of Leu and Ala residues (*top*), and of Leu, Ala, Val, Ile, Gly, Phe, Thr, Ser, Met, Tyr, Trp, Pro, Asn and Arg residues with position-defined constraints (*bottom*). Non-glycosylated protein bands are indicated by an empty dot; singly and doubly glycosylated proteins are indicated by one or two black dots, respectively.

To be able to directly compare the predicted insertion efficiencies to insertion into the mammalian ER, some of the computationally designed sequences were analysed using a well-established *in vitro* assay for quantifying the efficiency of membrane integration of designed TM sequences into dog pancreas rough microsomes (RMs)^8^. The host protein (Lep) consists of two TM segments (H1 and H2) connected by a cytoplasmic loop (P1) and a large C-terminal domain (P2), and inserts into ER-derived RMs with both termini located in the lumen (Fig. 2A). The designed sequence (“TM-tested”) was engineered into the luminal P2 domain and flanked by two acceptor sites (G1 and G2) for *N*-linked glycosylation. The engineered glycosylation sites can be used as membrane insertion reporters because G1 will always be glycosylated due to its native luminal localization, but G2 will be glycosylated only upon translocation of the analyzed region through the microsomal membrane. A singly glycosylated construct in which TM-tested is inserted into the membrane has a molecular mass ~2.5 kDa higher than the molecular mass of Lep expressed in the absence of microsomes; the molecular mass shifts by ~5 kDa upon double glycosylation (i.e. membrane translocation of the TM-tested).

We measured membrane insertion efficiencies of systematically designed sequences GGPG-X_11–23_-GPGG, in which the flanking tetrapeptides are included to insulate the central computed stretches of different lengths (11 to 23 residues long) from the surrounding sequence in the Lep model protein. The insertion efficiency was calculated on the basis of the fractions of singly (*f*_ig_) and doubly (*f*_2g_) glycosylated forms by using *p*_*i*_ = *f*_1g_/(*f*_1g_ + *f*_2g_) determined from quantitative analyses of SDS-PAGE gels. Examples of SDS-PAGE gels showing the translation products of non-position defined leucine and alanine (LA) stretches 11, 13, 15 and 17 residues long and position-defined LAVIGFTSMYWPNR stretches of 17, 19, 21 and 23 residues long are shown in Fig. 2B.

**Figure 3:**
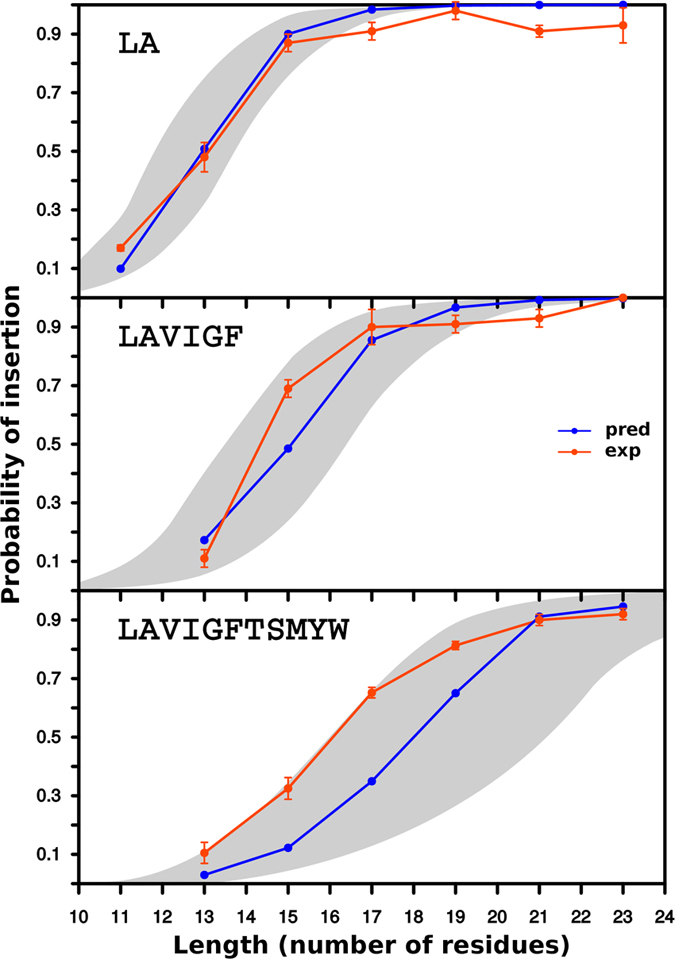
Predicted and experimental *p*_*i*_ values of sequences including the most prevalent amino acid residues in TM helices. Upper panel: Computationally designed leucine and alanine sequences of different lengths (*l*). The predicted values for each given sequence are shown in blue and the measured values obtained for three independent experiments in orange. The gray area represents the predictions of the *p*_*i*_ values for the 500 sequences between percentiles 0.25 and 0.75 of the total population (1,000 computed sequences, see Fig. S2). Central panel: Similar to upper but the computationally designed TM sequences contained leucine, alanine, valine, isoleucine, glycine and phenylalanine. Bottom panel: Similar to upper but including, in addition to the hydrophobic, the more prevalent polar and aromatic residues in TM segments. See Supplementary Information Table 2 for details on the TM-tested sequences used.

Figure 3 shows plots of the probability of membrane insertion as a function of TM length for series of sequences that included the more prevalent amino acid residues in TM helices. Hydrophobic Leu and Ala account for more than one quarter (27.7%) of all amino acids in TM helices, and together with Val, Ile, Gly and Phe constitute the bulk of the amino acids embedded into the hydrophobic core of the membrane, accounting for almost two thirds (64.4%) of all amino acids in this membrane region^7^. The grey area corresponds to the 500 sequences between percentiles 0.25 and 0.75 of the 1,000 predicted *p*_*i*_ values (SI Fig. S2). The figure also shows the probability of insertion for the experimentally measured sequences (orange line) as well as their particular predicted values (blue line) (see SI Table S2 for details on the TM segments analyzed).

**Figure 4:**
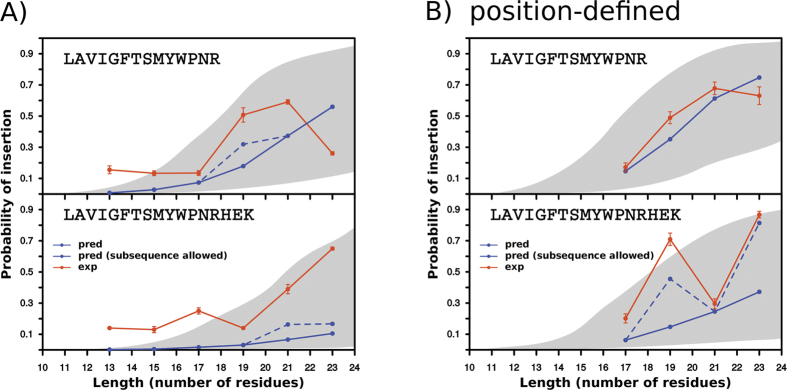
Predicted and experimental *p*_*i*_ values of computationally designed TM sequences including polar and charged amino acid residues. Experimental and predicted values are shown as in Fig. 3. (**A**) Computationally designed TM sequences were constrained only by amino acid composition constraints. (**B**) Computationally designed TM sequences were constrained both by amino acid composition and distribution along the helix. See Supplementary Information Table 2 for details on the TM-tested sequences used.

As expected, the inclusion of less prevalent amino acid residues in the designed TM sequences increased the variability of the predictions (compare the grey areas in Figs 3 and 4A) for all sets of sequence lengths. Moreover, the differences between the predictions and the experimental measurements were larger for some sequences (Fig. 4A). Next, we introduced constrains in our computational designs derived from the position-defined distributions found in native TM helices^7^. The introduction of the amino acid position constrains in our computational algorithm increased both the predicted as well as the experimentally measured *p*_*i*_ values for the TM sequences containing less abundant (polar) residues (Fig. 4B). Nevertheless, we noticed that in some cases the differences between the predicted and experimental values were large. In these cases, we re-ran the ΔG Predictor algorithm but this time the algorithm was allowed to identify subsequences (i.e., with lower ΔG estimated values). The new predictions, in all cases, approached the experimental values (Fig. 4A,B, dashed lines), reinforcing that biological membranes can adapt to accommodate sequences harboring deviations from canonical hydrophobic regions.

**Figure 5:**
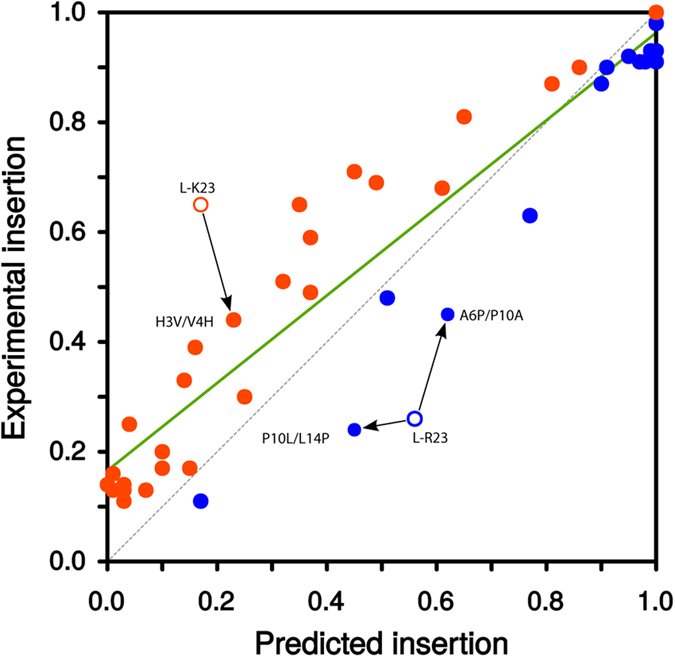
Correlation between experimentally measured and predicted *p*_*i*_ values. For each sequence analyzed, predicted values higher than the experimental ones (i.e., below the grey dashed line) are shown in blue, whereas experimental values higher than the predicted ones (i.e., above the grey dashed line) are shown in orange. The correlation between the experimental and predicted insertion probabilities is indicated by a green line. Outliers are shown as empty circles and the results of their mutated sequences (that is, P10L/L14P, A10P/P10A and H3V/V4H) are indicated by arrows.

Considering the complexity of the biological system, the two sets of *p*_*i*_ values are well correlated (Fig. 5): the linear fit has a slope of 0.80 with an *r* value of 0.93. Interestingly, the only outliers (highlighted as empty dots) are the longer sequences designed without position-dependent constrains, which include polar/charged residues. A closer look to the first of these sequences (L-K23, empty orange dot) revealed the presence of a histidine and glutamate residues (Table 1). This construct inserted experimentally much more efficiently than predicted by the ΔG algorithm. Given the 3.6-residue periodicity of an ideal α-helix, an intrahelical charge pair would be expected for this (*i, i* + 3) His-Glu pair. To test this hypothesis, we swapped the histidine residue with its neighboring valine residue (L-K23 H3V/V4H, Table 1) generating an (*i, i* + 2) periodicity for the His-Glu pair and likely precluding intrahelical pairing by orienting the two side-chains toward opposite faces of the helix. Noticeably, this mutant resulted in a slightly increased predicted *p*_*i*_ value but consistently diminished experimental insertion efficiency. The combined effect of these data improved the correlation between the experimental and prediction values for the mutant sequence (Fig. 5, arrow pointed dot), which has the same amino acid composition as the L-K23 sequence. These results support the idea that intra-helical salt-bridge formation between residues located on the same face of a TM helix (*i, i* + 3) may reduce the free energy of membrane partitioning^9^,^10^, whereas the presence of His and Glu on opposite faces of the helix (*i, i* + 2) is unfavorable and lowers the ER translocon membrane insertion efficiency.

Next, we analyzed the L-R23 (Fig. 5, empty blue dot) sequence. In this case the predicted value suggests a higher propensity to insert than the measured experimental value. Inspection of L-R23 sequence (Table 1) highlighted the presence of a proline residue in a central position within the hydrophobic region (−2 relative to the center, negatively labeled positions indicate extra-cytoplasmic face). In general, the presence of proline residues in an α-helix generates a constrained Φ rotamer at the position of the proline, the loss of a hydrogen bond donor and the appearance of steric clashes between the proline cyclic side chain and the peptide backbone. In the case of TM helices, all these effects may eventually increase the polarity of the carbonyl groups of the TM helix at the positions three and four residues N-terminal of the proline location^11^, reducing insertion efficiency^12^.

Based on the L-R23 construct, we made two different mutants with the proline residue placed at different positions. In particular, we compared the insertion efficiency of positioning the proline roughly one helical turn (4 residues) towards the N or C terminus by swapping mutagenesis. When the proline residue was moved towards the C terminus (+2 position, mutant P10L/L14P) the experimental *p*_*i*_ value remained very similar to the one obtained for the original sequence (see Table 1), whereas the construct in which the proline was moved four residues towards the N terminus (−6 position, mutant A6P/P10A) was inserted more efficiently into the ER (Table 1). The different effect observed for these two mutants can be explained by the different location of the proline residue in relation to the midpoint of the TM segment. Hence, in the case of P10L/L14P mutant (+2 position) the distortions produced by the proline occur around the center of the membrane plane where the system is probably more sensitive to distortions. On the contrary, in the case of A6P/P10A mutant (−6 position) the presence of the proline closer to the interface would locate the unsatisfied carbonyl group in a less hydrophobic environment^13^,^14^, probably reducing the free energy of membrane partitioning.

## Discussion

In this study, we have systematically explored how the amino acid composition and positioning affect the efficiency of membrane insertion capacity of computationally designed TM segments, using both prediction and experimental measurements. We used a microsomal *in vitro* expression system to examine the translocon insertion efficiency of chosen examples of the designed sequences. To generate the sequences we took advantage of the calculated distributions of amino acids from our previous structure-based statistical analysis of TM helices^7^.

Prior work showed that polyleucine sequences of 9–10 residues were efficiently inserted by the ER translocon into microsomal membranes^5^,^15^ and by the *E. coli* translocon^16^. However, TM segments in natural membrane proteins are not made exclusively of leucines. To expand our knowledge towards natural membrane proteins, we have designed and analyzed large sets of sequences with amino acid compositions that become more and more like the natural ones. Using the ΔG Prediction Server we found that 12–14 consecutive hydrophobic residues is about the minimum required for insertion into biological membranes through the ER translocon for highly hydrophobic sequences composed by leucine, alanine, valine and isoleucine (L-I series), which account for almost half (47.8%) of amino acid residue composition in TM helices. Sequences containing less prevalent (more hydrophilic) amino acid residues in TM segments have to be longer to efficiently insert into the membrane. Not surprisingly, there is a correlation between amino acid abundance in TM helices and hydrophobicity (SI Fig. 3), which explains the need for an increased sequence length compensating the lower hydrophobicity. However, this effect can be partly balanced by taking into account amino acid position-dependent contributions. Thus, when this last parameter was included in our computational designs, the predictions for the insertion efficiency of sequences harboring polar and charged residues increased significantly (Fig. 1C).

The results of our experimental assay using microsomal membranes (Figs 3 and 4) mirrored the ΔG algorithm predictions. Our data reinforces the accepted idea that there is an unfavorable free energy associated with locating hydrophilic residues in the hydrophobic core of the membrane. However, this effect can be reduced by allocating non-hydrophobic residues close to the polar headgroup region of the lipids at the membrane interface^17^, as well as by engaging polar residues in salt-bridge pair formation^9^,^18^. This is in line with our analysis of specific sequences with experimental/predicted *p*_*i*_ values that deviate from a linear correlation (Fig. 5). One of these outlier sequences (L-K23) was inserted by the translocon surprisingly efficiently compared with its ΔG Predictor value (Table 1). We suggest that the stability of L-K23 TM helix within the lipid membrane is derived from intra-helical electrostatic and/or salt-bridge interaction between the histidine and the glutamic acid side chains positioned at (*i, i* + 3) periodicity. In TM helices, intra-helical charge pairs within the same helix have been reported for appropriately spaced (*i, i* + 3 and *i, i* + 4), oppositely charged residues^9^,^10^. We support this hypothesis by locating the His-Glu pair at (*i, i* + 2) periodicity, which is non-compatible with intra-helical electrostatic and/or salt-bridge interaction. This mutation strongly reduced the experimental insertion efficiency. As expected, ionizable histidine and glutamic acid residues are present in TM helices at a low frequency level (1.7% and 1.6%, respectively). Nevertheless, among the 792 TM helices included in our database, 84 helices (10.6%) contained both amino acid residues in their sequence, and 15 of these helices present the His-Glu pair at (*i, i* + 3) periodicity. Approximately, only one fourth of these His-Glu pairs (4) are partly exposed to the lipid face, whereas the rest are buried in the protein interior, emphasizing the necessity to shield the polarity of this interaction from the hydrophobic environment of the membrane core. Charged and polar residues can face a high energetic barrier when inserting into a biological membrane. Nevertheless, positive and negative charges within the same^9^,^10^ and different^19^,^20^ TM helices can interact with each other, thereby drastically reducing this barrier. In fact, these interactions can occur in the proximity of the translocon^21^,^22^, where a strict coupling of correct tertiary structure formation and membrane insertion can be achieved^23^.

The other outlier sequence (L-R23) displayed a completely different behavior, since in this case the experimentally measured *p*_*i*_ value was lower than the predicted value (Fig. 5). This sequence contains a proline residue at a nearly central position in the TM segment. Proline is rarely found in the middle of helices from soluble proteins because it results in distortion of the canonical helical geometry and loss of at least one backbone hydrogen bond^24^,^25^. However, proline residues are relatively common in TM helices^7^,^11^. This suggests that proline residues may be of particular structural and/or functional significance in membrane proteins, even though they invariably produce deviations from canonical helical structure^26^. To learn how the proline present in the L-R23 mutant reduces membrane insertion, we analyzed the insertion of a mutant with the proline residue positioned near the middle of the helix (P10L/L14P), and a mutant with the proline residue located near the N-terminus of the helix (A6P/P10A), both with the same amino acid composition. The A6P/P10A mutant inserted more efficiently than the original sequence, while the P10L/L14P sequence resulted in only minor changes in terms of membrane insertion. These results indicate that proline residues are not easily accommodated in the center of the helix. Structural studies have shown that proline substitutions at the end of a TM helix can be accommodated by movement of a small part of the helix, while proline substitutions in the middle can require more complex and difficult to accommodate structural changes^26^. Statistical analyses of TM helices show a similar pattern for proline residue distribution (SI Fig. S4). Altogether, these results indicate that the proline in the original sequence can diminish membrane insertion efficiency depending on the position along the TM segment.

In summary, our analysis of the membrane insertion of computationally designed TM sequences resulted in a good correlation between the values predicted by the ΔG Predictor and experimentally measured values. Nevertheless, our data indicate that some extra attention has to be paid to accommodate intra-helical salt-bridge formation and proline residues when designing short TM helices as building blocks of membrane proteins, a major challenge when engineering new membrane proteins to perform biomimetic functions.

## Methods

### Computational sequence design

All statistics of amino acid composition at the TM level as well as the positional level was derived from our previously published datasets^7^. Briefly, our dataset included a total of 170 non-redundant structures described in the MPtopo database^27^ containing 792 TM helices of length from 17 and 38 residues, which resulted in a total of 19,356 amino acids. The dataset was further parsed to compute the probability of a given amino acid to be included in a TM helix. Using our dataset, we generated a series of designed sequences of a given length by populating them with an increased number of amino acid types. First, we generated sequences with only Leu amino acid type (the most common in TMs) of lengths ranging from 9 to 25 residues. Next, we included the second most common amino acid type in TMs (that is, Ala) with its relative probability compared to Leu. Again, we generated 1,000 sequences of each length where the sequences were obtained by shuffling a string of L and A letters with the proportions defined in Supplementary Information Table S1. The same procedure was repeated each time including a new amino acid type until we had all 20. Next, we generated a set of sequences with both, amino acid propensity as well as positional effect by taking TMs residues and annotating their position with respected the central part of the TM helix. In such case, we computed the probability of an amino acid type in each of the positions of a TM starting from the central one (position 0) and increasing the position number as we approached the cytoplasmic side of the TM or a negative number as we approached the extracellular side of the TM. The designed sequences were built taking into account the position by selecting amino acids for each pool across the TM helices. Similarly to the no position-defined dataset, 1,000 sequences of each different length were generated for each amino acid type compositions.

### Prediction of the ΔG values and probability of insertion

All sets of 1,000 sequences of desired length, amino acid composition and non-positional or positional effect were used to generate a series of predicted insertion capacity scores, which were predicted using the experimentally based ΔG Prediction Server (http://dgpred.cbr.su.se/)^2^,^3^. The generated sequences and their ΔG values can be obtained in the provided Excel file (SI Table S3).

### Enzymes and chemicals

All enzymes as well as plasmid pGEM1, the TNT SP6 Quick Coupled System and rabbit reticulocyte lysate were from Promega (Madison, WI). ER rough microsomes from dog pancreas were from tRNA Probes (College Station, TX). [^35^S]Met were from Perkin Elmer. The restriction enzymes were purchased from Roche Molecular Biochemicals. The DNA purification kits were from Thermo (Ulm, Germany). All the oligonucleotides were purchased from Sigma-Aldrich (Switzerland).

### DNA manipulation

For experimental analysis of the computationally designed sequences, oligonucleotides encoding the computed hydrophobic (TM) regions were introduced into the P2 domain of *E. coli* leader peptidase (Lep). Tested sequences were constructed using two double-stranded oligonucleotides with 5′ phosphorylated overlapping overhangs at the ends. Pairs of complementary oligonucleotides were first annealed at 85 °C for 10 min followed by slow cooling to 30 °C, after which the two or three annealed double-stranded oligonucleotides were mixed, incubated at 65 °C for 5 min, cooled slowly to room temperature and ligated into the vector. Mutations at the designed TM segments were obtained by site-directed mutagenesis using the QuikChangee kit (Stratagene, La Jolla, California). All TM segment inserts and mutants were confirmed by sequencing of plasmid DNA.

### *In vitro* transcription and translation

Constructs in pGEM1 were transcribed and translated in the TNT SP6 Quick Coupled System (Promega). 75 ng DNA template, 0.5 μL ^35^S-Met (5 μC_i_) and 0.25 μL microsomes (tRNA Probes) were added at the start of the reaction, and samples were incubated for 90 min at 30 °C. Translation products were diluted in 50 μL of loading buffer and analyzed by SDS–PAGE. The gels were quantified using a Fuji FLA-3000 phosphoimager and Image Reader 8.1j software. The membrane-insertion probability of a given TM sequence was calculated as the quotient between the intensity of the singly glycosylated band divided by the summed intensities of the singly glycosylated and doubly glycosylated bands.

## Additional Information

**How to cite this article**: Baeza-Delgado, C. *et al*. Biological insertion of computationally designed short transmembrane segments. *Sci. Rep.* **6**, 23397; doi: 10.1038/srep23397 (2016).

## Supplementary Material

Supplementary Information

Supplementary Dataset 1

## Figures and Tables

**Table 1:**
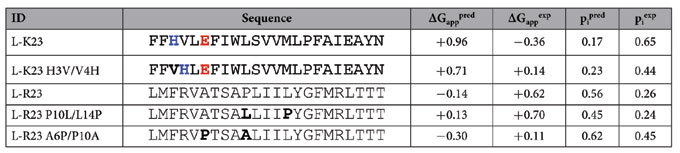
Thermodynamic cost of L-K23- and L-R23-derived TM segments integration.

The predicted and experimental (Δ*G*_*app*_) energetic cost in kcal/mol of the computationally designed TM segments. Negative values are indicative of TM disposition, while positive values indicate non-TM disposition. Charged residues studied are highlighted in color and mutated residues are shown in bold.
