# Supplementary material for: Biological insertion of computationally designed short transmembrane segments
Source: Sci Rep. 2016 Mar 18;6:23397. doi: 10.1038/srep23397 (PMC4796907; doi:10.1038/srep23397)
Supplement: Supplementary Information [file srep23397-s1.pdf]

Biological insertion of computationally designed short transmembrane segments

Carlos Baeza-Delgado [1] , Gunnar von Heijne [2] , Marc A. Marti-Renom [3,4,5] and Ismael Mingarro [1] *

[1] Departament de Bioquímica i Biologia Molecular, ERI BioTecMed, Universitat de València. E-46100 Burjassot, Spain

[2] Dept. of Biochemistry and Biophysics and Science for Life Laboratory, Stockholm University, 10691 Stockholm, Sweden.

[3] CNAG-CRG, Centre for Genomic Regulation (CRG), Barcelona Institute of Science and Technology (BIST), 08028 Barcelona, Spain

[4] Universitat Pompeu Fabra (UPF), 08002 Barcelona, Spain

[5] Institució Catalana de Recerca i Estudis Avançats (ICREA), 08010 Barcelona, Spain

Supplementary Information

Supplementary Figure 1

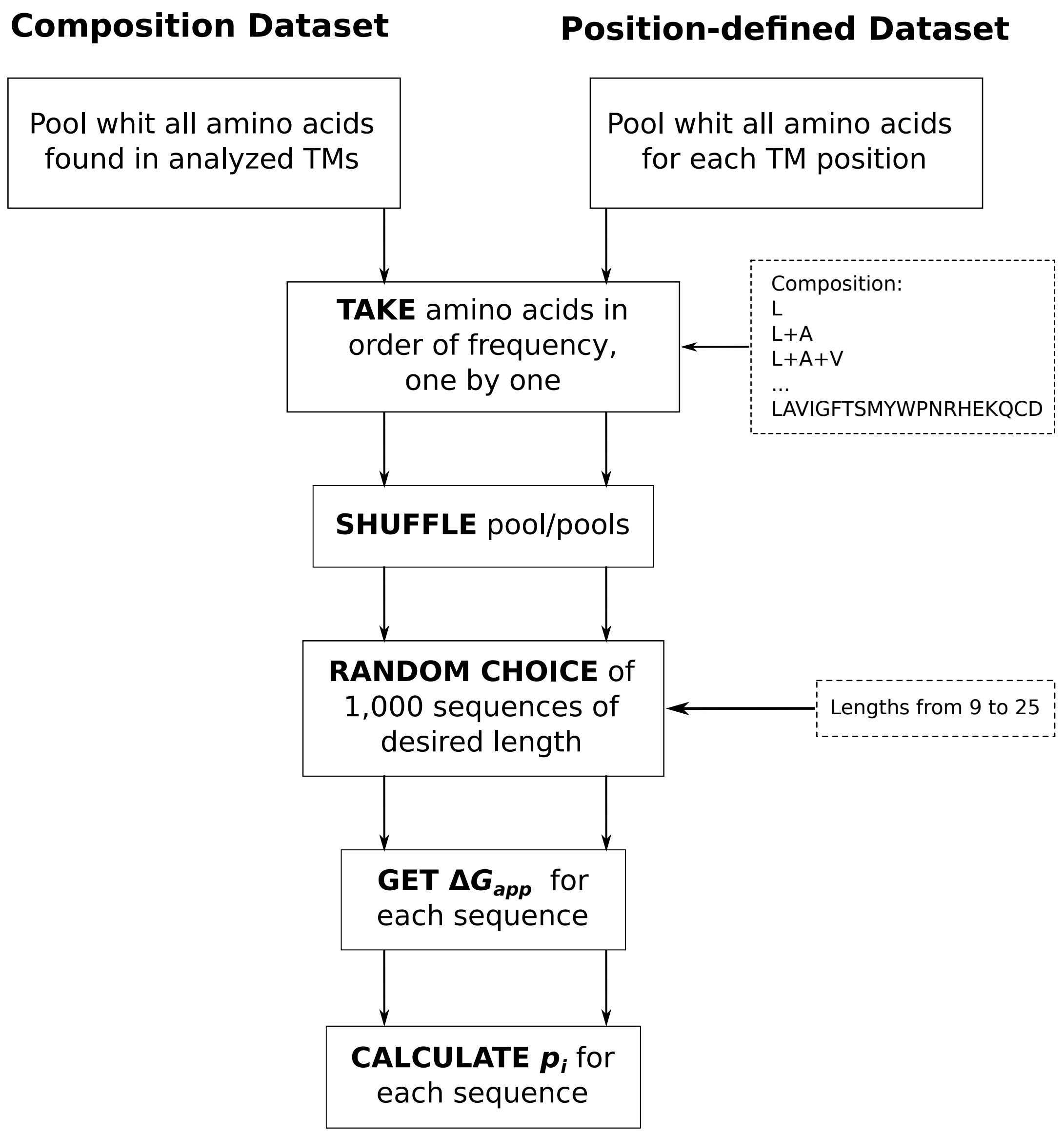

Figure S1. Computational flowchart for designing TM sequences as well as predicting their insertion probability using the experimentally based ΔG Prediction Server (http://dgpred.cbr.su.se).

Supplementary Figure 2 (L/A-derived sequences):

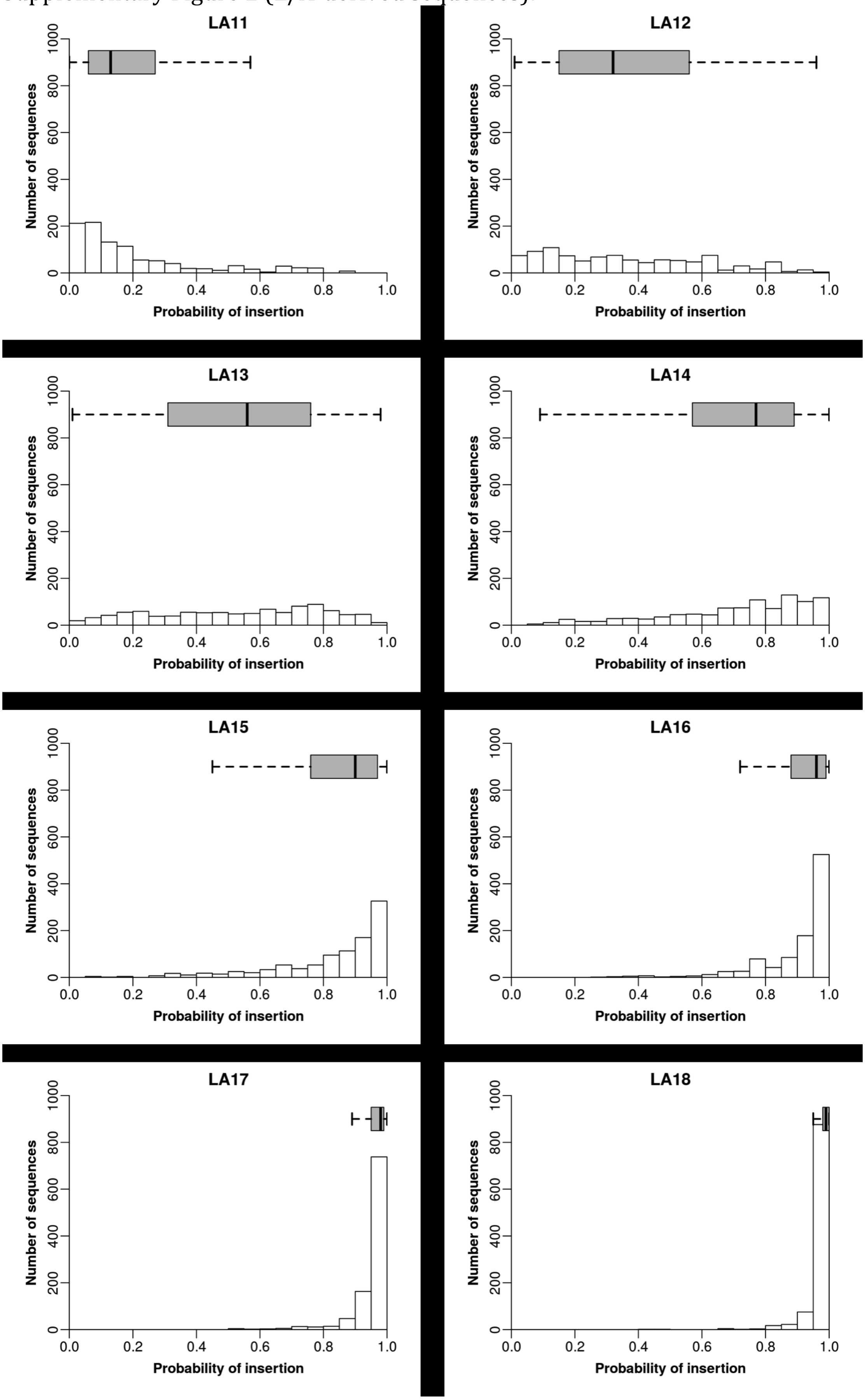

Supplementary Figure 2 (L/A/V/I/G/F-derived sequences):

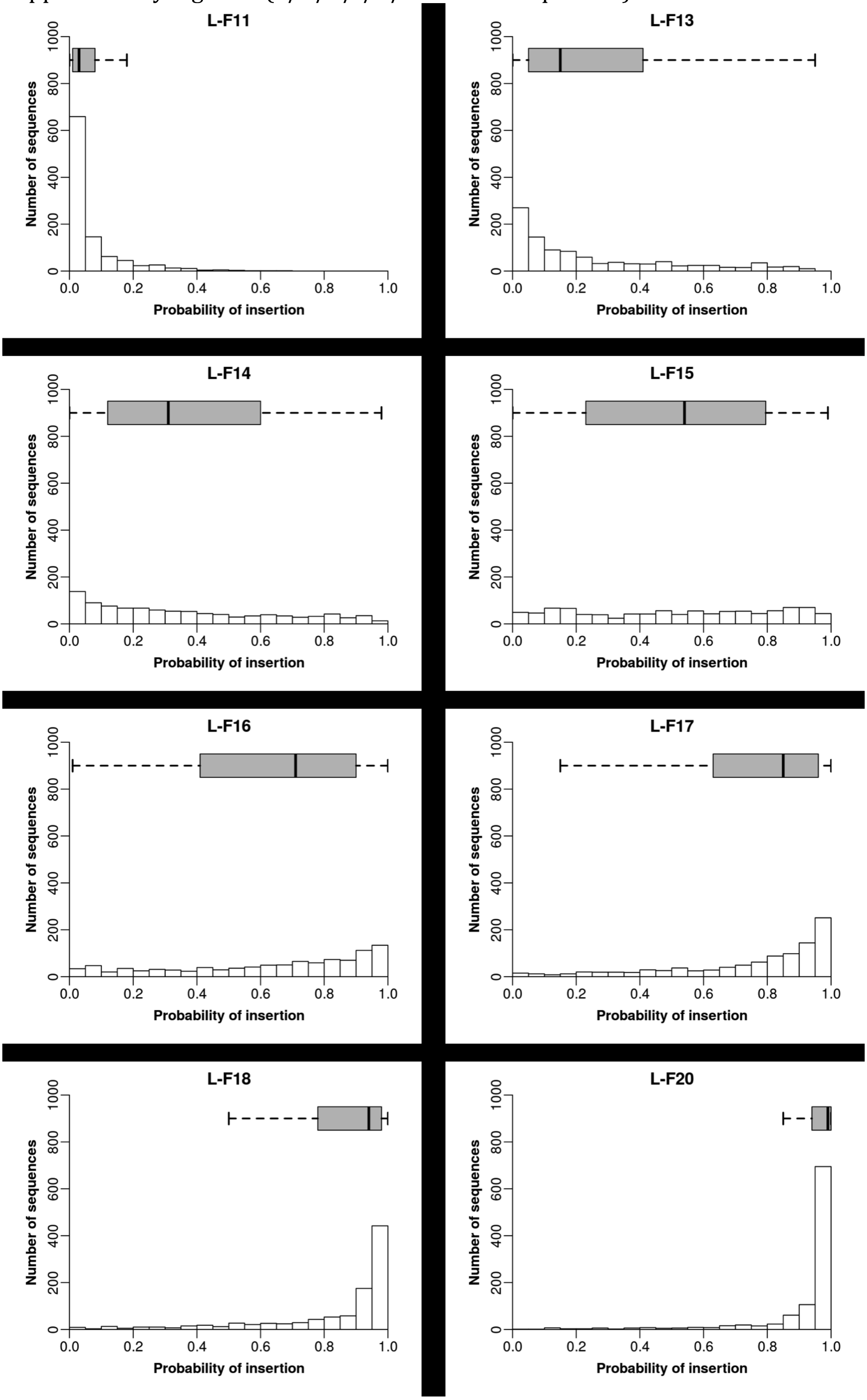

Supplementary Figure 2 (L/A/V/I/G/F/T/S/M/Y/W-derived sequences):

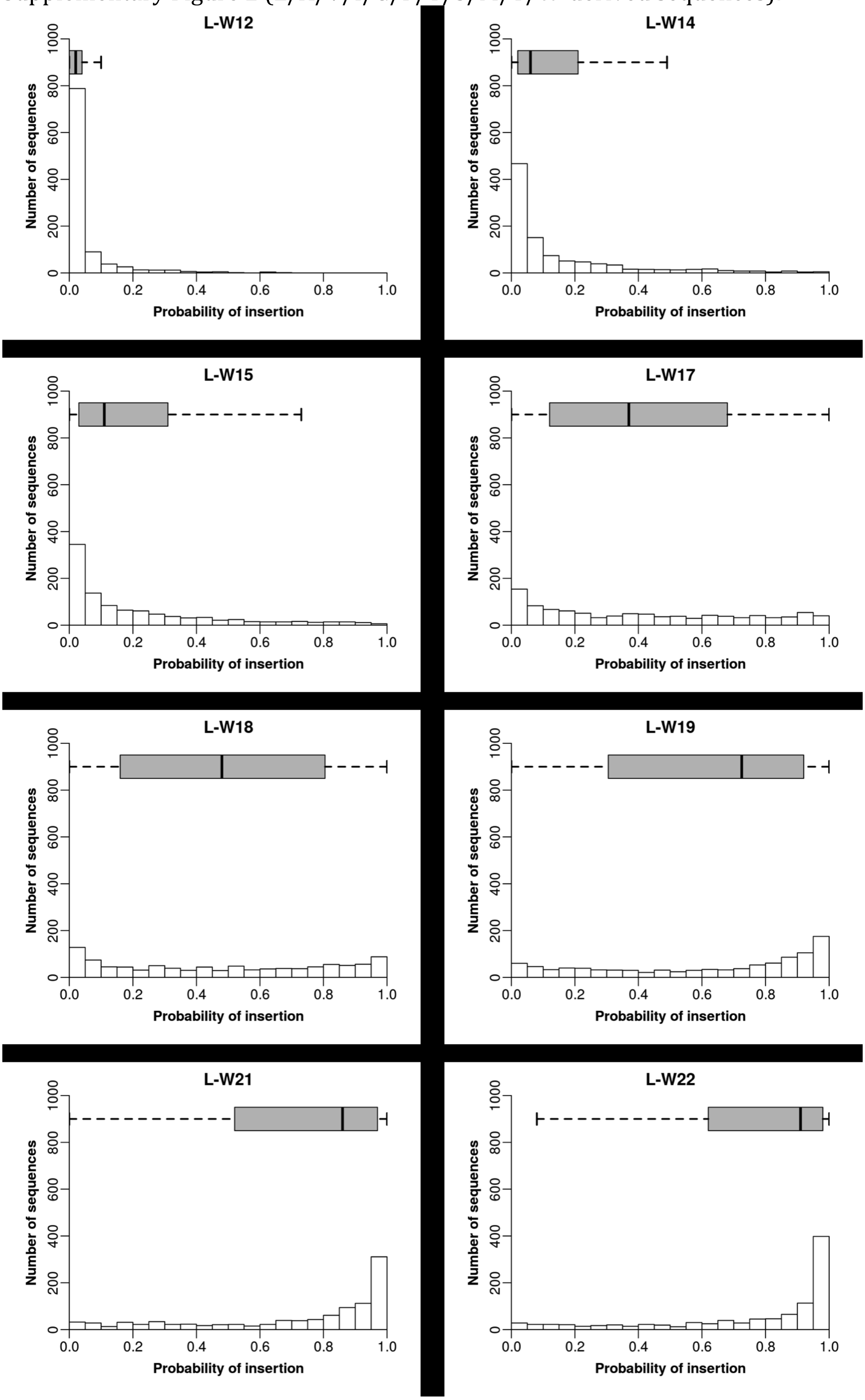

Suppl. Figure 2 (L/A/V/I/G/F/T/S/M/Y/W/P/N/R-derived sequences):

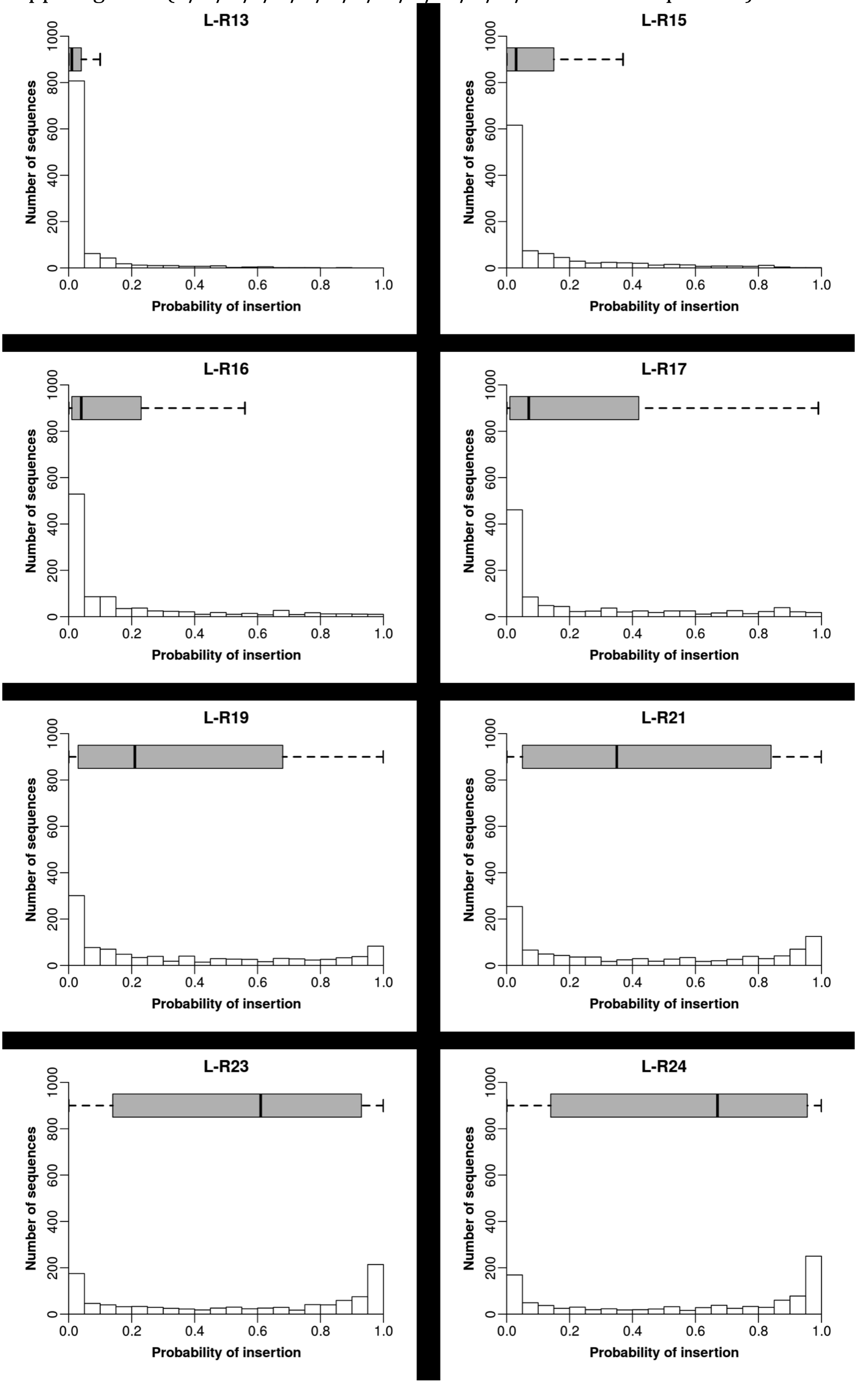

Suppl. Figure 2 (L/A/V/I/G/F/T/S/M/Y/W/P/N/R/H/E/K-derived sequences):

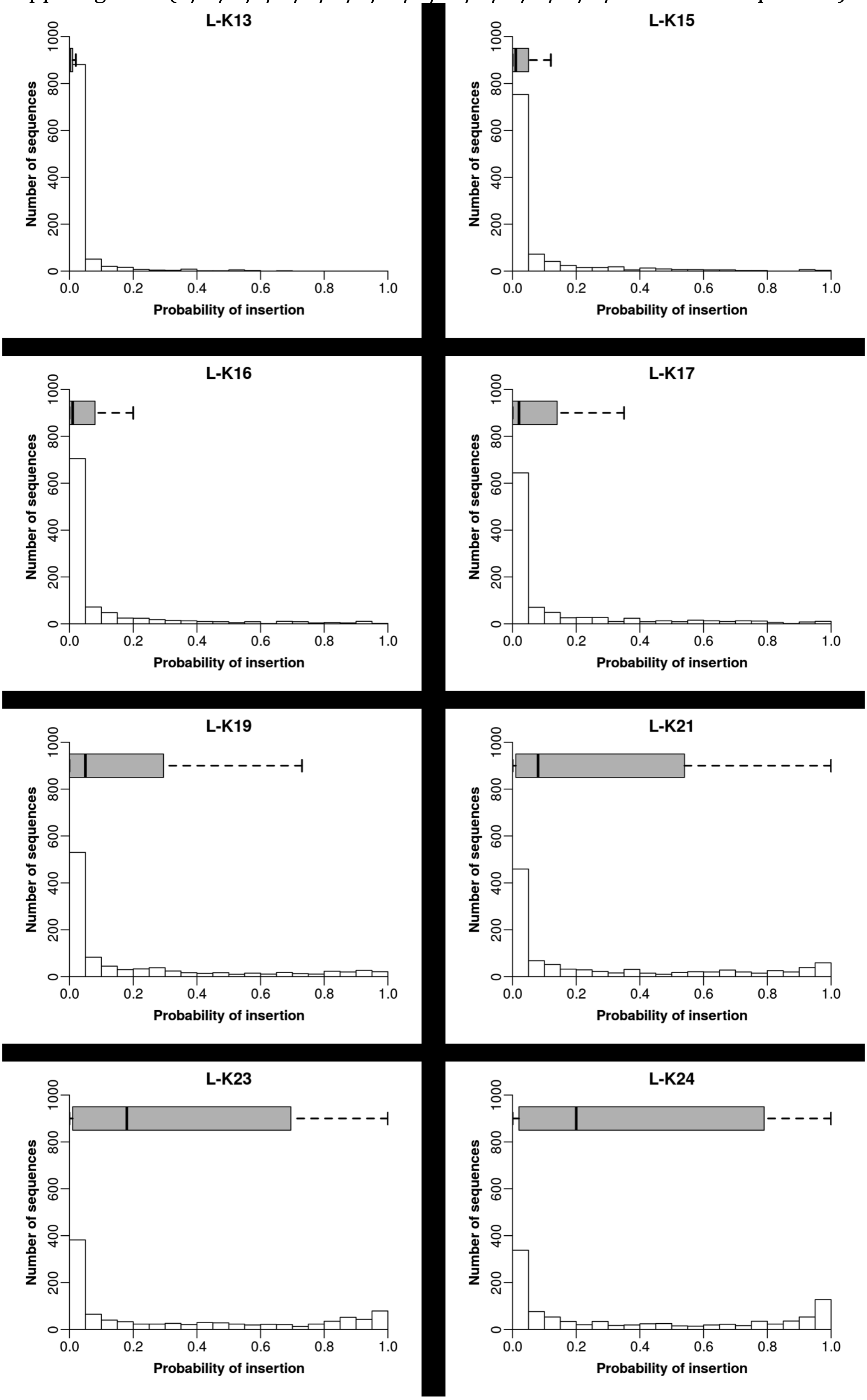

Suppl. Figure 2 (LR-derived position-defined sequences):

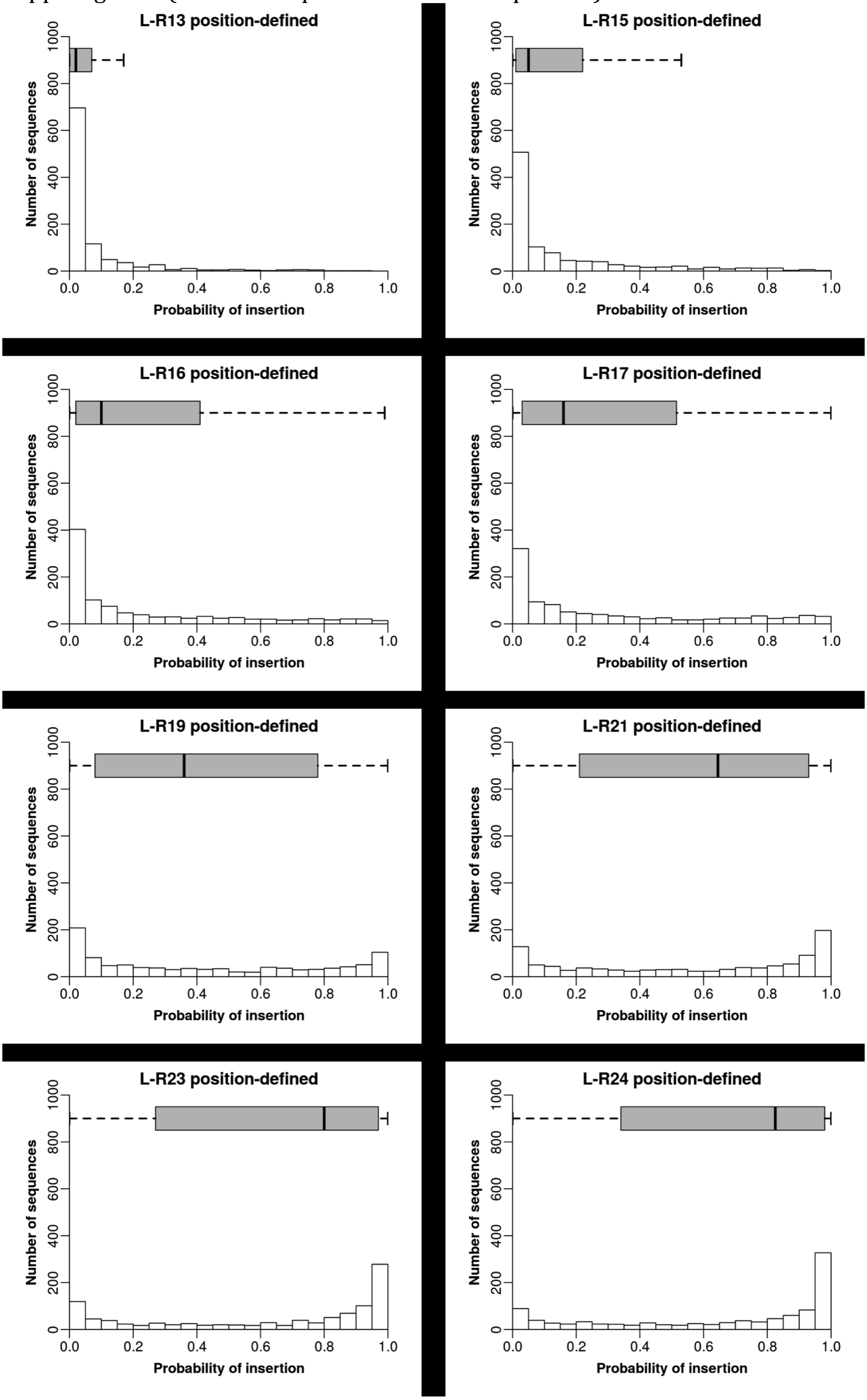

Suppl. Figure 2 (LK-derived position-defined sequences):

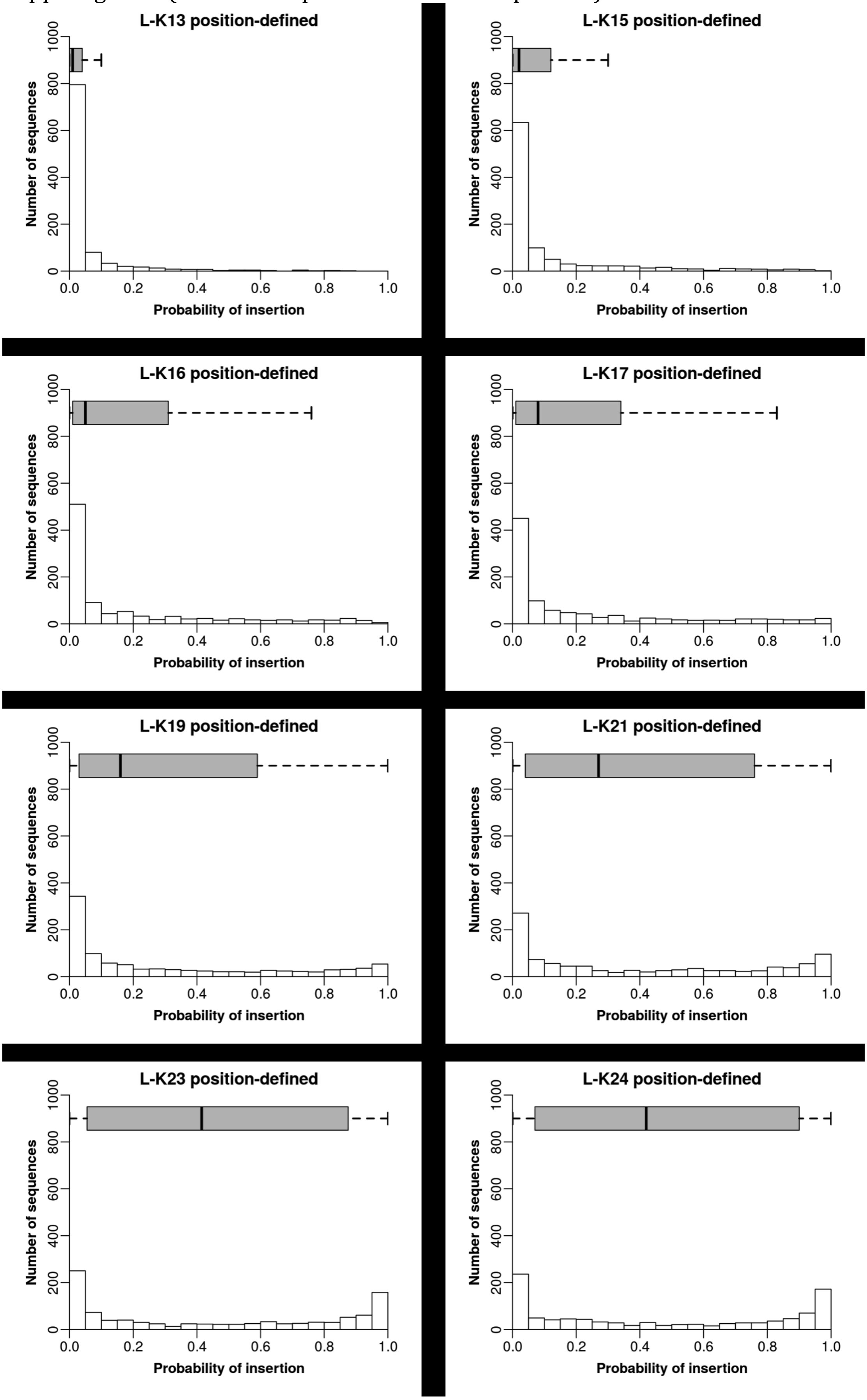

Figure S2. Distribution of sequences with predicted p_i values. Sequences were grouped in 0.05 p_i window range. The grey boxes correspond to the predicted p_i values for the 500 sequences between percentiles 0.25 and 0.75. Dotted lines correspond to the predicted p_i values for the 250 sequences with higher standard deviations on both sides.

Supplementary Figure 3

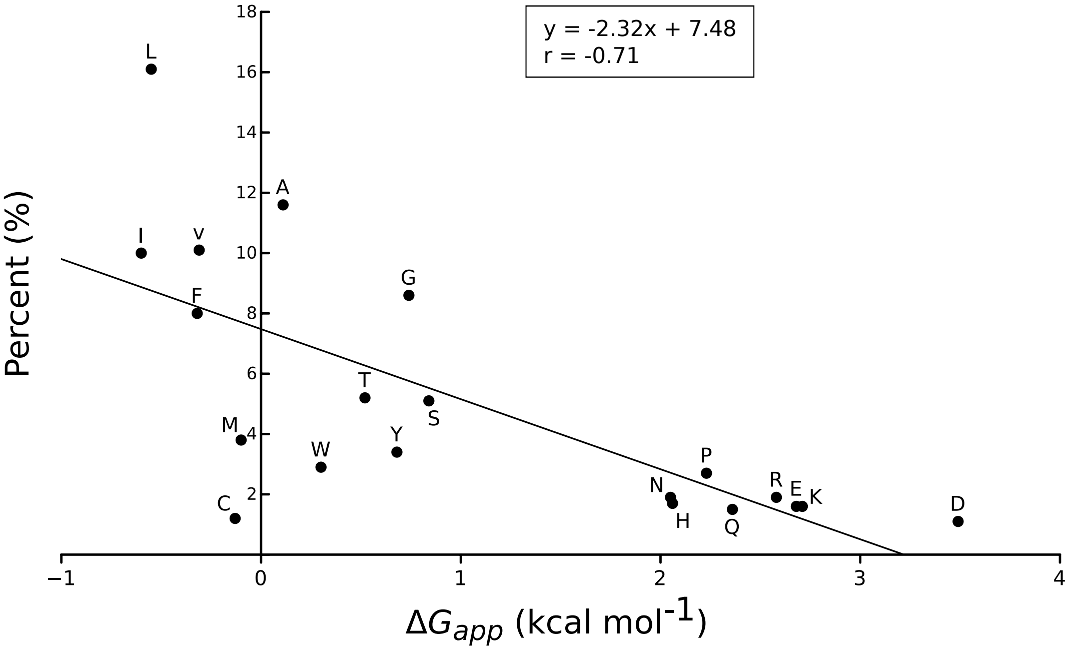

Figure S3. Correlation between the amino acid type prevalence in TM helices of membrane protein structures [1] and the biological free energy scale [2].

Supplementary Figure 4

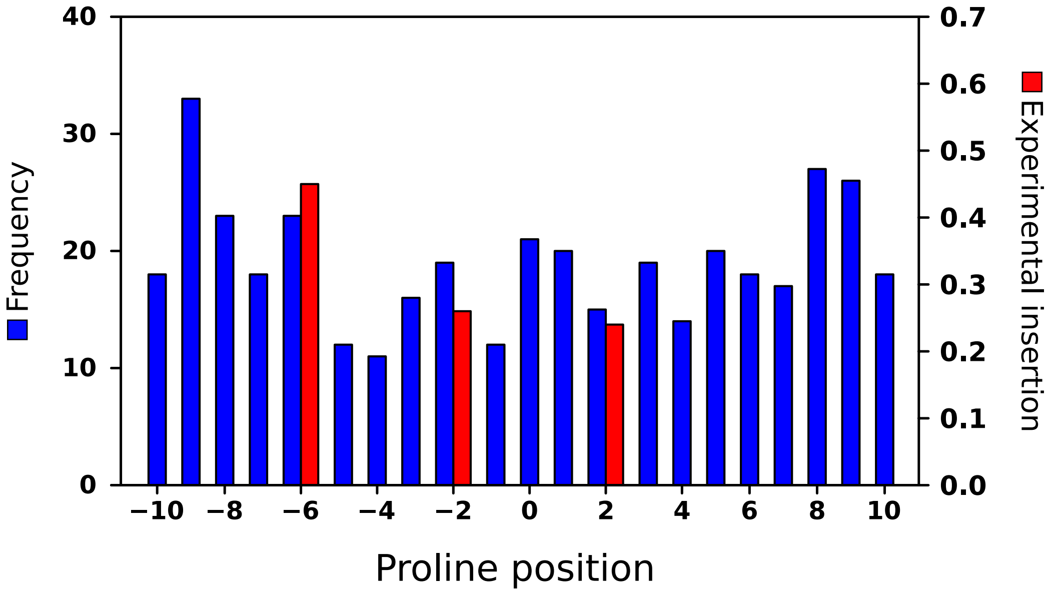

Figure S4. Proline positions along TM helices. The frequency of proline residues in TM helices is shown as a function of their position within the helices. Positively labeled positions indicate the cytoplasmic side of the membrane whereas negatively labeled positions are indicative of extra-cytoplasmic regions. The measured p_i values for the L-R23-derived sequences containing single proline residues are shown in red.

Table S1. Amino acid relative ratio values used in the computational calculations. We included one by one the 20 natural amino acids following the order of prevalence found in native proteins [1].

%	L	A	V	I	G	F	T	S	M	Y	W	P	N	R	H	E	K	Q	C	D
**L**	100.0	-	-	-	-	-	-	-	-	-	-	-	-	-	-	-	-	-	-	-
**LA**	58.2	41.8	-	-	-	-	-	-	-	-	-	-	-	-	-	-	-	-	-	-
**LAV**	42.6	30.6	26.8	-	-	-	-	-	-	-	-	-	-	-	-	-	-	-	-	-
**L-I**	33.6	24.2	21.2	21.0	-	-	-	-	-	-	-	-	-	-	-	-	-	-	-	-
**L-G**	28.5	20.5	18.0	17.8	15.3	-	-	-	-	-	-	-	-	-	-	-	-	-	-	-
**L-F**	24.9	17.9	15.7	15.6	13.4	12.4	-	-	-	-	-	-	-	-	-	-	-	-	-	-
**L-T**	23.1	16.6	14.5	14.4	12.4	11.5	7.5	-	-	-	-	-	-	-	-	-	-	-	-	-
**L-S**	21.5	15.5	13.6	13.4	11.5	10.7	7.0	6.8	-	-	-	-	-	-	-	-	-	-	-	-
**L-M**	20.5	14.7	12.9	12.8	11.0	10.2	6.6	6.5	4.8	-	-	-	-	-	-	-	-	-	-	-
**L-Y**	19.6	14.1	12.4	12.3	10.5	9.8	6.4	6.2	4.6	4.1	-	-	-	-	-	-	-	-	-	-
**L-W**	19.0	13.6	12.0	11.8	10.2	9.5	6.1	6.0	4.5	4.0	3.4	-	-	-	-	-	-	-	-	-
**L-P**	18.4	13.2	11.6	11.5	9.8	9.2	5.9	5.8	4.3	3.9	3.3	3.1	-	-	-	-	-	-	-	-
**L-N**	18.0	12.9	11.3	11.2	9.6	9.0	5.8	5.7	4.3	3.8	3.2	3.0	2.1	-	-	-	-	-	-	-
**L-R**	17.6	12.7	11.1	11.0	9.4	8.8	5.7	5.6	4.2	3.7	3.1	2.9	2.1	2.1	-	-	-	-	-	-
**L-H**	17.3	12.4	10.9	10.8	9.3	8.6	5.6	5.5	4.1	3.6	3.1	2.9	2.0	2.0	1.9	-	-	-	-	-
**L-E**	17.0	12.2	10.7	10.6	9.1	8.5	5.5	5.4	4.0	3.6	3.0	2.8	2.0	2.0	1.8	1.7	-	-	-	-
**L-K**	16.7	12.0	10.5	10.4	9.0	8.3	5.4	5.3	4.0	3.5	3.0	2.8	2.0	2.0	1.8	1.6	1.6	-	-	-
**L-Q**	16.5	11.8	10.4	10.3	8.8	8.2	5.3	5.2	3.9	3.5	2.9	2.7	1.9	1.9	1.8	1.6	1.6	1.5	-	-
**L-C**	16.3	11.7	10.2	10.2	8.7	8.1	5.3	5.1	3.8	3.4	2.9	2.7	1.9	1.9	1.8	1.6	1.6	1.5	1.2	-
**L-D**	16.1	11.6	10.1	10.0	8.6	8.0	5.2	5.1	3.8	3.4	2.9	2.7	1.9	1.9	1.7	1.6	1.6	1.5	1.2	1.1

Table S2. Predicted and measured ΔG (kcal/mol) and p_i values for all sequences in the study. All hydrophobic sequences were flanked by insulating glycine-proline regions (GGPG-X_n-GPGG), where X represent the designed sequences of *n* residues length.

ID	Sequence	ΔG_{app}^{pred}	ΔG_{app}^{exp}	p_i^{pred}	p_i^{exp}
LA11	LAALLLAAALL	1.31	0.92	0.10	0.17
LA13	LLALALALLAALL	-0.02	0.05	0.51	0.48
LA15	LALLLALAALLALAL	-1.30	-1.15	0.90	0.88
LA17	AALALLLALLALALALL	-2.43	-1.36	0.98	0.91
LA19	LALALALAALALLLLALLA	-3.52	-4.23	1.00	1.00
LA21	LALLLAALLALALLLLAAAAL	-4.55	-1.38	1.00	0.91
LA23	ALLAALLLALLLLAALALAALAL	-5.52	-2.69	1.00	0.99
L-F13	FFFIFGGVILIAA	0.93	1.27	0.17	0.10
L-F15	AAIALLGLAVGLFLF	0.04	-0.48	0.49	0.69
L-F17	AAAFAIAGLFIAIVLIF	-1.05	-1.43	0.86	0.92
L-F19	AIILFLLVLIGVGVLAAGV	-1.99	-1.37	0.97	0.91
L-F21	VGIFVGLIFLAVLGIIALLLG	-2.90	-1.55	0.99	0.93
L-F23	IILVIAGAFVVGVFAVFVLVLLF	-3.63	-5.45	1.00	1.00
L-W13	MLTVATIFISLGF	2.07	1.29	0.03	0.10
L-W15	GFVAYVAMIAWLLIG	1.08	0.43	0.14	0.32
L-W17	VSFVGITVAFFWLVFTM	0.37	-0.37	0.35	0.65
L-W19	AMISVLVGWVMVLLFFAGT	-0.37	-0.87	0.65	0.81
L-W21	MTLFMIIMLLAMYAWAGGLVG	-1.38	-1.31	0.91	0.90
L-W23	IVLLAGLALYSGIVAVVTIFMWM	-1.70	-1.46	0.95	0.92
L-R13	LMRVFAVVLGNVI	2.93	1.01	0.01	0.15
L-R15	LPLLWASVITAGTVL	2.12	1.11	0.03	0.13
L-R17	RWGTTAYFMLAVIAAPF	1.50	1.11	0.07	0.13
L-R19	PLLRPFFFTLVTVLAYMAIV	0.45	-0.02	0.32	0.51

ID	Sequence	ΔG_{app}^{pred}	ΔG_{app}^{exp}	p_i^{pred}	p_i^{exp}
L-R21	LFMAPVWYLVGNLITALLIYL	0.31	-0.22	0.37	0.59
L-R23	LMFRVATSAPLIILYGFMRLTTT	-0.14	0.62	0.56	0.26
L-K13	IFFEWLVGLGMGI	3.64	1.09	0.00	0.14
L-K15	VIEIVLIYHLVPIWI	3.12	1.14	0.01	0.13
L-K17	LFFFAVSLKLAWTMFVP	1.96	0.66	0.04	0.25
L-K19	PFIIWAASLSIGAKLSYAW	2.04	1.05	0.03	0.14
L-K21	MIFLLLLPMAILHRSPLLVGK	0.97	0.27	0.16	0.39
L-K23	FFHVLEFIWLSVVMLPFAIEAYN	0.95	-0.36	0.17	0.65
L-R17 position-defined	LIVLFVRMLLAVLGLNG	1.05	1.29	0.15	0.10
L-R19 position-defined	LAVITLAIAWFMSIFGAYP	0.31	0.62	0.37	0.26
L-R21 position-defined	RIWSILYIALTWTFILAGASR	-0.27	0.29	0.61	0.38
L-R23 position-defined	AAYIYSINLFAFVTMVLFARLPA	-0.71	0.29	0.77	0.38
L-K17 position-defined	SVALMFSLVGMYIFGLH	1.27	1.20	0.10	0.12
L-K19 position-defined	WVEPTVYLIITFLALLRVK	0.11	0.25	0.45	0.40
L-K21 position-defined	YWIEVPSVVIITVAAFGVLSF	0.67	0.99	0.25	0.16
L-K23 position-defined	TWKIISGLVFLALFIWGMYPSEA	-0.87	-0.02	0.81	0.51
L-K23 H3V/V4H	FFVHLEFIWLSVVMLPFAIEAYN	0.71	0.14	0.23	0.44
L-R23 P10L/L14P	LMFRVATSALLIIPYGFMRLTTT	0.13	0.70	0.45	0.24
L-R23 A6P/P10A	LMFRVPTSAALIILYGFMRLTTT	-0.30	0.11	0.62	0.45

Bibliography

1 Baeza-Delgado, C., Marti-Renom, M. A. & Mingarro, I. Structure-based statistical analysis of transmembrane helices. *Eur Biophys J* **42**, 199-207, doi:10.1007/s00249-012-0813-9 (2013).

2 Hessa, T. *et al*. Recognition of transmembrane helices by the endoplasmic reticulum translocon. *Nature* **433**, 377-381 (2005).
